# Transcriptomic and functional analyses of 3D placental extravillous trophoblast spheroids

**DOI:** 10.1038/s41598-019-48816-8

**Published:** 2019-08-30

**Authors:** Michael K. Wong, Mishquatul Wahed, Sarah A. Shawky, Anna Dvorkin-Gheva, Sandeep Raha

**Affiliations:** 1 Graduate Program in Medical Sciences, McMaster University, Hamilton, Ontario L8N 3Z5 Canada; 2 Department of Pediatrics, McMaster University, Hamilton, Ontario L8N 3Z5 Canada; 3 McMaster Immunology Research Centre, McMaster University, Hamilton, ON L8N 3Z5 Canada

**Keywords:** Cell invasion, Reproductive biology

## Abstract

Placental extravillous trophoblast (EVT) invasion is essential in establishing proper blood supply to the fetus during pregnancy. However, traditional 2D *in vitro* systems do not model the *in vivo* invasion process in an anatomically-relevant manner. Our objectives were to develop a 3D spheroid model that would allow better emulation of placental invasion *in vitro* and to characterize the transcriptomic and functional outcomes. HTR8/SVneo EVT cells were self-assembled into 3D spheroids using ultra-low attachment plates. Transcriptomic profiling followed by gene set enrichment and gene ontology analyses revealed major global transcriptomic differences, with significant up-regulations in EVTs cultured as 3D spheroids in canonical pathways and biological processes such as immune response, angiogenesis, response to stimulus, wound healing, and others. These findings were further validated by RT-qPCR, showing significant up-regulations in genes and/or proteins related to epithelial-mesenchymal transition, cell-cell contact, angiogenesis, and invasion/migration. A high-throughput, spheroid invasion assay was applied to reveal the dynamic invasion of EVTs away from the spheroid core into extracellular matrix. Lastly, lipopolysaccharide, dexamethasone, or Δ^9^-tetrahydrocannabinol exposure was found to impact the invasion of EVT spheroids. Altogether, we present a well-characterized, 3D spheroid model of EVT invasion and demonstrate its potential use in drug and toxin screening during pregnancy.

## Introduction

The human placenta is a transient organ that forms the interface between the mother and fetus during pregnancy^1^. One of its key functions during early pregnancy is invasion and migration into the maternal decidua^2,3^. Proper invasion is critical to the remodeling of maternal spiral arteries into high capacitance, low resistance vessels that supply oxygen and nutrients to the fetus^4^. The primary placental cell type responsible for regulating invasion is the extravillous trophoblast (EVT), which bears the ability to migrate away from the solid trophoblast columns of the anchoring chorionic villi and invade into the maternal decidua^5^. This process is highly regulated by a variety of physiochemical factors (*e*.*g*. oxygen, growth factors, nutrients, extracellular matrix proteins), and dysregulated EVT invasion can conversely result in compromised placentation and an inability to properly support both the mother and fetus^6^. Interestingly, pregnancy-related diseases like preeclampsia and placenta accreta have been strongly associated with altered EVT invasion and spiral artery remodeling, both poorly understood pathological outcomes of impaired trophoblast function^6^. However, our ability to better understand the cellular and molecular underpinnings of these placental diseases may be limited, in part, by the unrepresentative nature of current two-dimensional (2D) *in vitro* systems.

Accumulating evidence in other organ systems and cancer models suggest that culturing cells in three-dimensions (3D) can provide more anatomically- and physiologically-relevant results compared to traditional 2D monolayer cultures^7^. While some researchers have begun to culture placental cells as 3D spheroids or organoids^8–12^, our overall understanding of the transcriptomic changes and functional outcomes associated with its formation remains preliminary. Characterization studies contrasting novel 3D spheroids against 2D monolayers are especially lacking – yet, these are necessary to justify its use and advancement, as the theorized advantages (*e*.*g*., enhanced cell-cell contact, physiological function, etc.^7^) are not verified in placental *in vitro* cultures. Moreover, there are no spheroid models to date that directly and specifically mimic the trophoblast column of the anchoring chorionic villi, and demonstrate functional EVT invasion and migration away from its core as seen *in vivo*^13^. To address this, we develop a self-assembling, 3D spheroid culture system of the trophoblast column and EVT invasion, and use a transcriptome-wide microarray and bioinformatics approach to characterize the global gene expression profiles against traditional 2D monolayers. We further apply a high-throughput, spheroid invasion assay to measure the actual invasion of the 3D EVT spheroids into extracellular matrix in real-time, and assess the impact of various drugs and compounds.

## Results

### Extravillous trophoblast cells self-assembled into 3D spheroids with high viability over eight days

**Figure 1:**
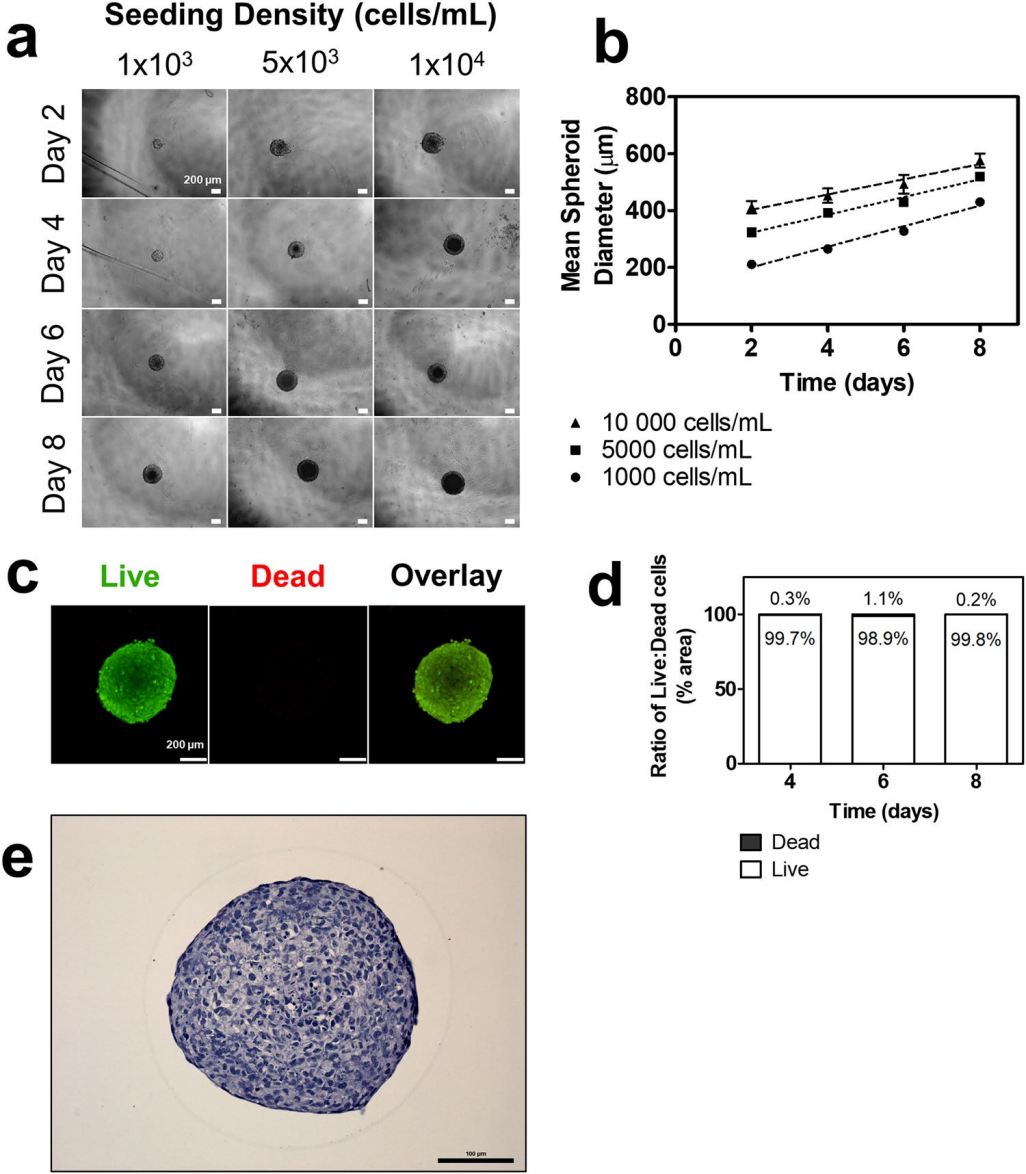
Self-assembly, growth, and viability of 3D EVT spheroids over eight days. (**a**) EVTs seeded at three different densities form spheroids that increase in size over eight days. 4x magnification. Scale bar indicates 200 µm. (**b**) Mean spheroid diameter over time across three seeding densities. (**c**) Immunofluorescent images of live and dead stain using calcein AM and ethidium homodimer-1, respectively. Green colour indicates live cells. Red colour indicates dead cells. 10x magnification. Scale bar indicates 200 µm. (**d**) Ratio of surface area of live to dead cells at days four, six, and eight. (**e**) Representative image of H&E staining of a spheroid cross-section. Scale bar indicates 100 µm. n = 3.

HTR8/SVneo EVT cells seeded in ultra-low attachment plates at densities of 1,000, 5,000, and 10,000 cells/mL all self-assembled into spheroids within two days with average diameters of 211.1 µm, 323.7 µm, and 411.6 µm, respectively (Fig. 1a). Spheroid diameter increased at rates of 36.05 ± 2.01, 31.26 ± 1.73 and 26.65 ± 3.47 µm/day over eight days, respective to the seeding densities, and maintained structural integrity throughout (Fig. 1a,b). Spheroids seeded at 5,000 cells/mL were used in subsequent experiments as they maintained mean diameters of less than 500 µm over eight days, above which has been frequently characterized with necrotic cores^14^. Indeed, live and dead staining using calcein AM and ethidium homodimer-1, respectively, confirmed that spheroids maintain high viability (98.9–99.9% live cells) over eight days (Fig. 1c,d). Hematoxylin and eosin (H&E) staining of cross-sections also revealed that the spheroids exhibit solid cores (Fig. 1e), resembling the solid structural anatomy of trophoblast columns *in vivo*^15^.

### Distinct transcriptomic profiles in EVTs cultured as 3D spheroids and 2D monolayers

**Figure 2:**
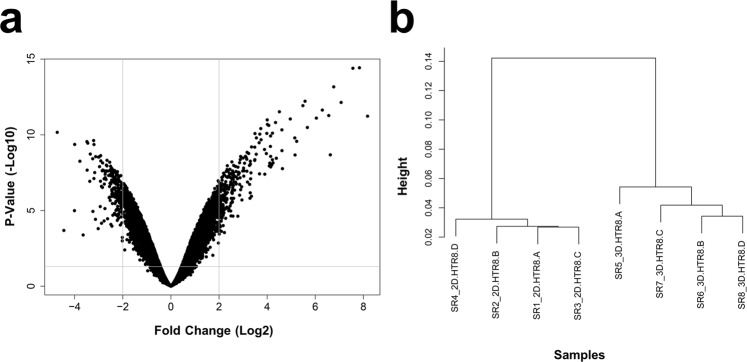
Transcriptome profiling of global gene expression changes in EVTs cultured as 3D spheroids compared to 2D monolayers. (**a**) Volcano plot comparing fold change (Log2) to p-value (-Log10) of differentially expressed genes measured using Clariom S Human microarray (Thermo). Significance determined by p < 0.05 and absolute fold change ≥2. (**b**) Dendrogram demonstrating clustering of the samples by similarity of transcriptome profiles. Four samples were analyzed per group, with different letters at the end of the name indicating a distinct sample.

To understand how global gene expression profiles were changed in EVTs cultured as 3D spheroids compared to 2D monolayers, we analyzed 21,448 genes via Clariom S Human transcriptome profiling microarray (Thermo). A total of 4,562 gene probe sets were found to be differentially expressed in 3D spheroids compared to 2D monolayers, with 2,327 genes up-regulated and 2,235 genes down-regulated (FDR p-value < 0.05; absolute fold change ≥2; Fig. 2a). Cluster dendrogram analyses demonstrated distinct grouping between samples from 3D spheroid and 2D monolayer groups and a large height distance to convergence, suggesting highly different global transcriptome profiles between the two groups (Fig. 2b). The short height distance to convergence seen for samples within each of the two groups suggests similar transcriptome profiles within each group (Fig. 2b).

### Gene set enrichment analysis of canonical pathways and biological processes

**Figure 3:**
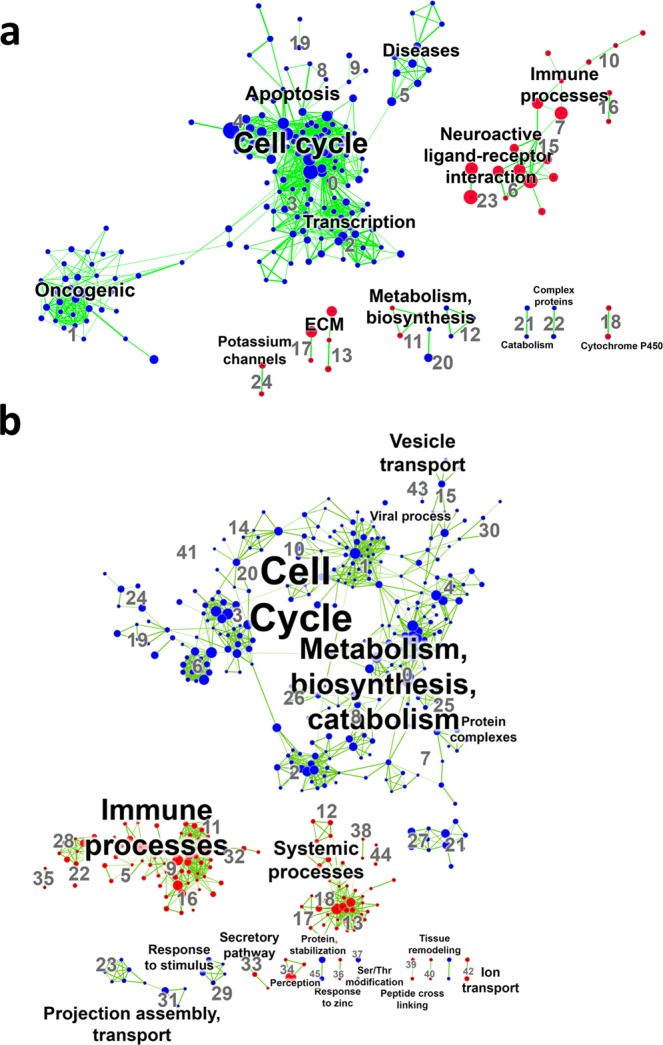
Enrichment maps of GSEA canonical pathways and biological processes comparing EVTs cultured as 3D spheroids and 2D monolayers. (**a**) Visualization of results of canonical pathway analyses and (**b**) biological process analyses. Every node (dot) represents a module of enriched genes in a particular pathway or process, with the size of the node representing number of genes. Red nodes indicate up-regulation in 3D spheroids compared to 2D monolayers and blue nodes represent down-regulation. Every edge (green line) represents overlap of genes between two pathways or processes, with the thickness of the line representing number of genes overlapping. Font size of annotated classification reflects number of modules within that classification group. Significance determined by FDR p-value < 0.05 and absolute fold change ≥2.

We next performed gene set enrichment analysis (GSEA) on the transcriptome microarray data to identify the enriched canonical pathways in extravillous trophoblasts cultured as 3D spheroid compared to 2D monolayers (Fig. 3a). The top fifteen most significantly enriched canonical pathway terms in EVTs cultured as 3D spheroids included categories: “neuroactive ligand-receptor interaction”, “immune processes, diseases, defensins”, “signaling events”, and “ECM” (FDR p < 0.05; Supplementary Table S1). The top fifteen most significantly down-regulated pathway terms in 3D spheroids included categories: “diseases”, “transcription, HIV life cycle”, “cell cycle”, and “cell cycle, apoptosis” (FDR p < 0.05; Supplementary Table S2). Full list of canonical pathway modules, categories, and terms may be found in Supplementary Table S3.

We also performed GSEA to identify the enriched biological processes in 3D spheroids compared to 2D monolayers (Fig. 3b). The top fifteen most significantly enriched biological process terms in EVTs cultured as 3D spheroids included categories: “immune processes”, “perception”, “chemotaxis”, “peptide cross linking”, “systemic processes”, and “response to zinc” (FDR p < 0.05; Supplementary Table S4). The top fifteen most significantly down-regulated biological process terms in 3D spheroids included categories: “protein complexes”, “metabolism, biosynthesis, catabolism”, “cell cycle”, and “transcription” (FDR p < 0.05; Supplementary Table S5). Full list of biological process modules, categories, and terms may be found in Supplementary Table S6.

### Gene ontology analysis of biological processes

**Figure 4:**
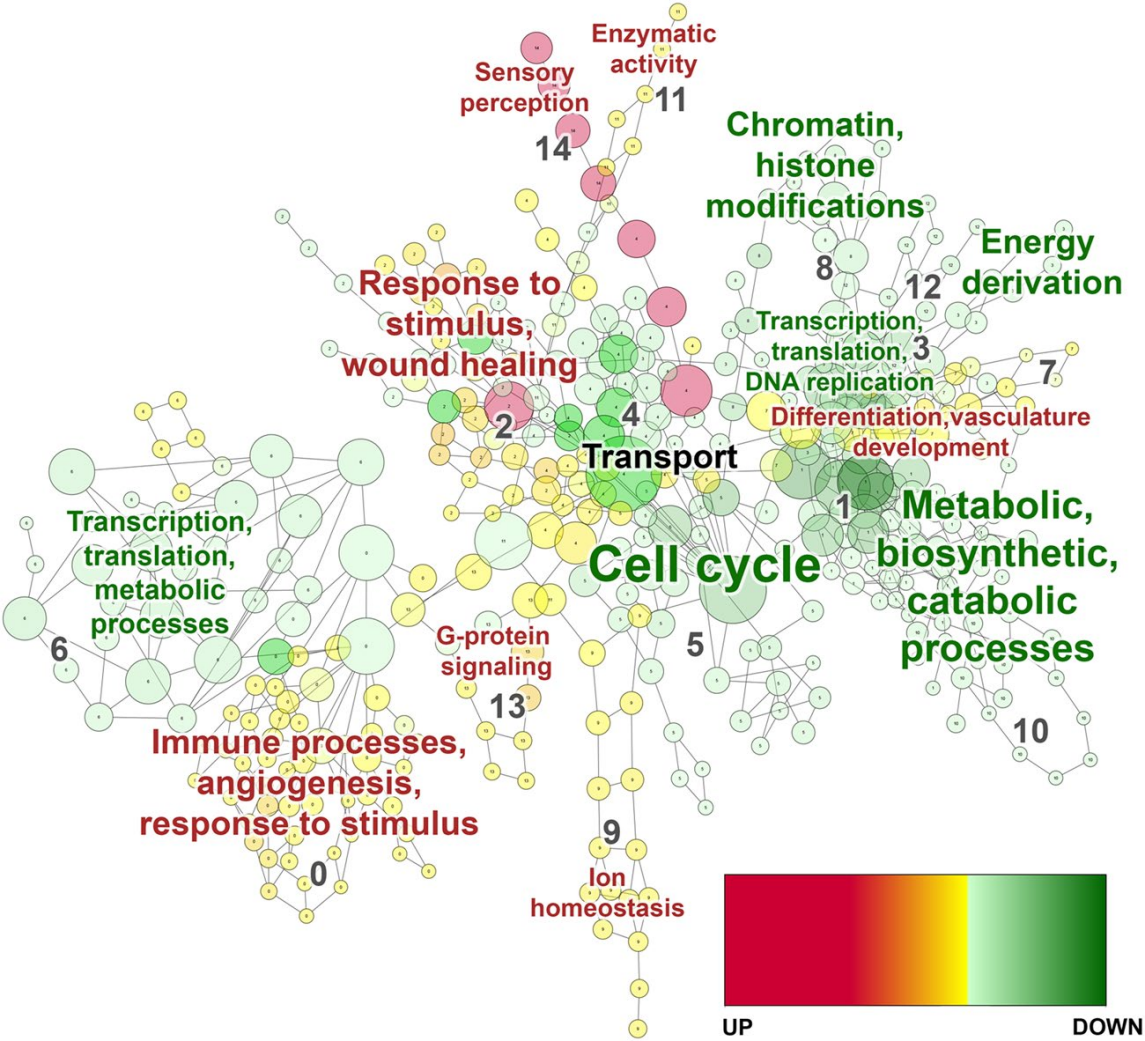
Gene ontology analysis map of biological processes comparing EVTs cultured as 3D spheroids and 2D monolayers. Visualization of results of biological process analyses. Every node (circle) represents a module of overrepresented genes in a particular process, with the size of the node representing number of genes. Nodes with red to yellow colour gradients indicate strong to weak up-regulation, respectively. Nodes with dark to light green colour gradients indicate strong to weak down-regulation, respectively. Every edge (line) represents overlap of genes between two pathways or processes. Red font colour indicates an up-regulated category in 3D spheroids compared to 2D monolayers, green font indicates down-regulated, and black font indicates a category with an even number of terms regulated in each direction. Font size of annotation reflects number of modules within that category. Significance determined by FDR p-value < 0.05 and absolute fold change ≥2.

In parallel, we performed gene ontology (GO) analysis on the transcriptome microarray data to elucidate the over-represented biological processes based on differential expression (Fig. 4). The top fifteen most significantly up-regulated biological process terms in EVTs cultured as 3D spheroids compared to 2D monolayers included categories: “sensory perception”, “transport”, “response to stimulus, wound healing”, “G-protein signaling”, and “immune processes, angiogenesis, and response to stimulus” (FDR p < 0.05; Supplementary Table S7). The top fifteen most significantly down-regulated biological process terms in spheroids compared to monolayers included categories: “metabolic, biosynthetic, catabolic processes”, “transcription, translation, DNA replication”, and “cell cycle” (FDR p < 0.05; Supplementary Table S8). Full list of GO biological process categories, terms, and gene lists may be found in Supplementary Table S9.

### Increased expression of markers related to epithelial-mesenchymal transition (EMT), cell-cell contact, angiogenesis, and invasion in EVTs cultured as 3D spheroids

**Figure 5:**
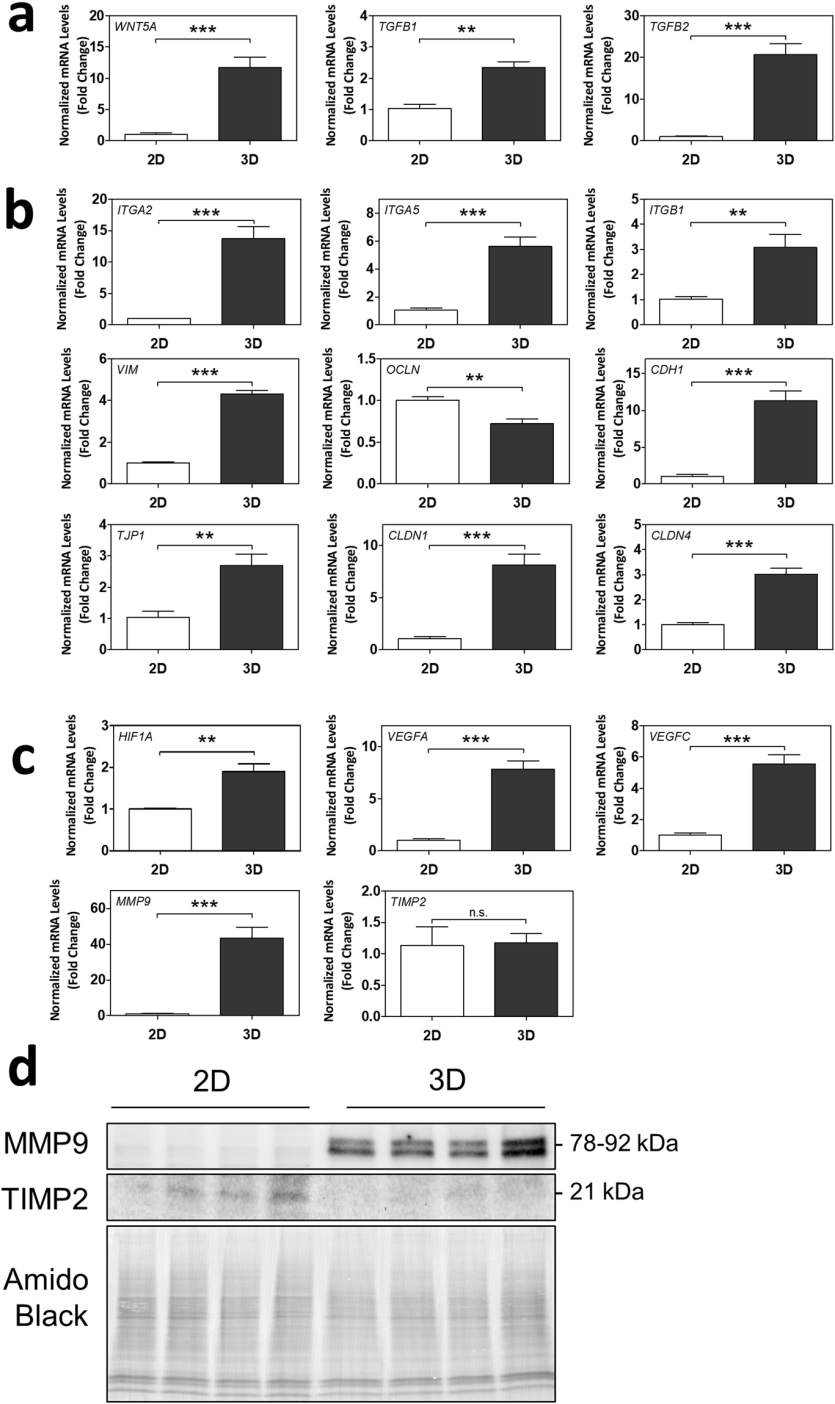
3D spheroids exhibit differential mRNA and protein expression of markers of epithelial-mesenchymal transition (EMT), cell-cell contact, angiogenesis, and invasion/migration. Normalized mRNA levels of (**a**) *WNT5A*, *TGFB1*, *TGFB2*, (**b**) *ITGA2*, *ITGA5*, *ITGB1*, *VIM*, *OCLN*, *CDH1*, *TJP1*, *CLDN1*, *CLDN4*, (**c**) *HIF1A*, *VEGFA*, *VEGFC*, *MMP9*, and *TIMP2*, as measured using RT-qPCR. Significant differences between groups determined by unpaired t-test; n = 4. Significant differences between means were indicated by **(p < 0.01) or ***(p < 0.001). Non-significant differences indicated by n.s. (**d**) Cropped blots of MMP9 and TIMP2 protein bands as detected by Western blot, and Amido Black staining of total protein. n = 4. Membrane was cut below 63 kDa and top half used for the MMP9 blot. Remaining membrane was cut above 25 kDa and lower half used for the TIMP2 blot. Whole blots with protein ladders found in Supplementary Fig. S1.

We were particularly intrigued by the up-regulated biological process categories of “immune processes, angiogenesis, response to stimulus”, “response to stimulus and wound healing”, and “differentiation and vasculature development” in 3D spheroids, given the important role played by EVTs in placental invasion and vascular remodeling (FDR p < 0.05; Supplementary Table S9). We first investigated changes in markers in the EMT pathway via RT-qPCR, as EMT of trophoblasts closely underlies the initiation of placental invasion^7,16^. mRNA levels of upstream EMT markers *WNT5A*, *TGFB1*, *and TGFB2* were significantly increased in 3D spheroids compared to 2D monolayers (p < 0.01 for all; Fig. 5a). Further downstream, integrin (*ITG*)*A2*, *ITGA5*, *ITGB1*, and vimentin (*VIM*) mRNA levels were increased in 3D spheroids compared to 2D, and occludin (*OCLN*) mRNA levels were decreased (p < 0.01 for all; Fig. 5b). Contrastingly, E-cadherin (*CDH1*) and zona occludens-1 (*TJP1*) mRNA levels were significantly increased in 3D spheroids (p < 0.01 for all; Fig. 5b), whereas they are typically repressed during EMT^16^. This may be explained in part by the fact that *CDH1* and *TJP1* were also markers of cell-cell contact and tight junction formation, which is promoted by 3D culture^7^. Claudin (*CLDN*)*1* and *CLDN4* mRNA levels, additional markers of tight junctions, were also significantly increased in 3D spheroids (p < 0.001 for all; Fig. 5b).

We further investigated placental angiogenesis and invasion gene markers shared across several of the biological process categories listed above. We found significant up-regulations in mRNA levels of *HIF1A*, *VEGFA*, *VEGFC*, and *MMP9* in EVTs cultured as 3D spheroids compared to 2D monolayers (p < 0.01 for all genes; Fig. 5c). mRNA levels of *TIMP2* remained unchanged (Fig. 5c). Protein levels of MMP9 were also increased in EVTs cultured as 3D spheroids, and protein levels of TIMP2 decreased (Fig. 5d; Whole blots provided in Supplementary Fig. S1). Altogether, this suggests that EVTs cultured as 3D spheroids exhibit enhanced expression of EMT activation, cell-cell contact, and tight junction formation in association with increased expression of angiogenesis and invasion.

### EVT spheroids exhibit dynamic invasion when embedded into ECM

**Figure 6:**
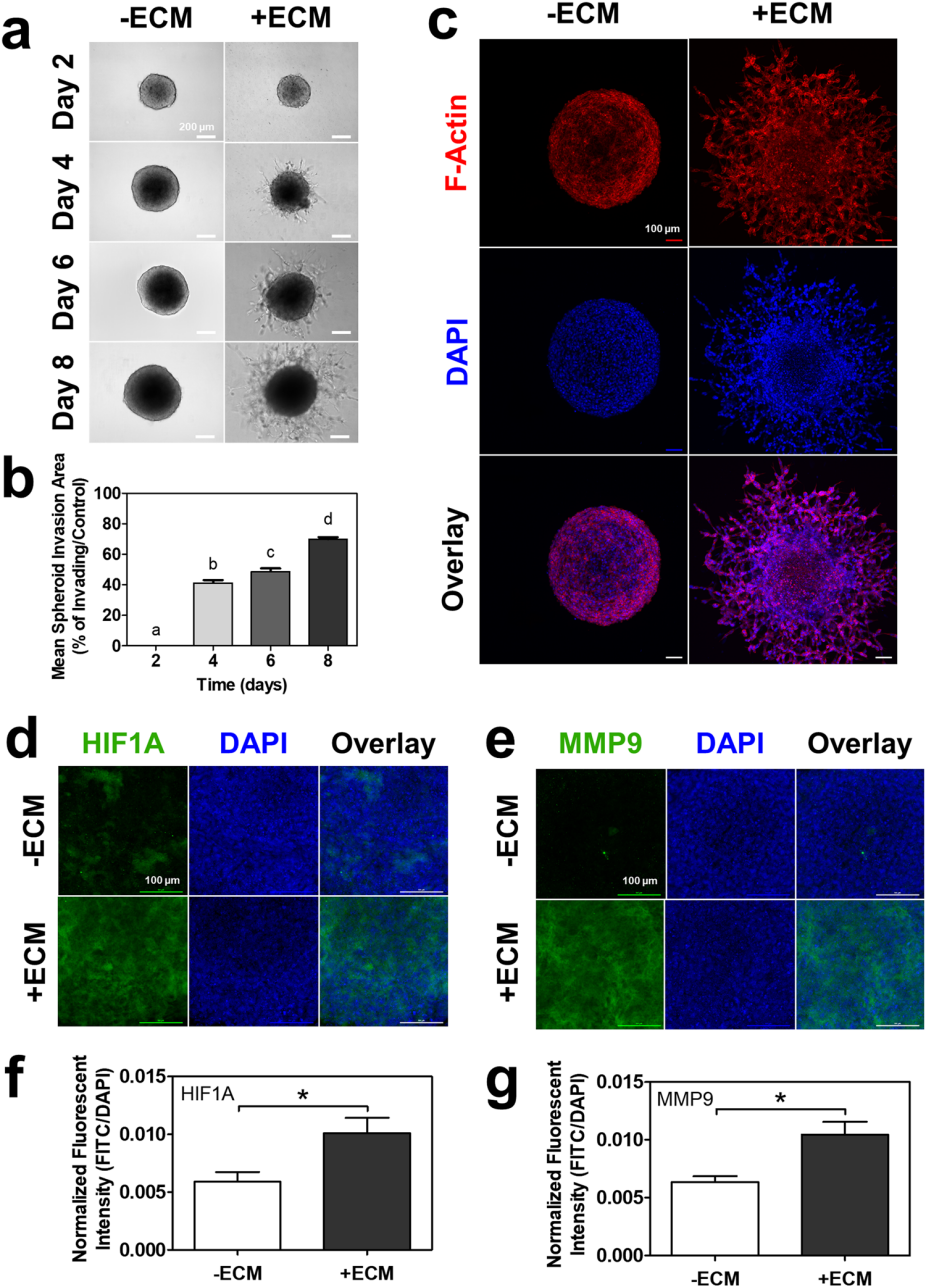
EVT spheroids exhibited continuous invasion into ECM over eight days. (**a**) Brightfield images of EVT spheroids without or with ECM over eight days. 10x magnification. Scale bar indicates 200 µm. (**b**) Histogram of mean spheroid invasion area percentage over eight days. Significant differences between groups determined by one-way ANOVA followed by Tukey’s post-test; n = 6. Significant differences between means as determined by post-tests were indicated by different letters. (**c**) Immunofluorescent confocal images of EVT spheroids without or with ECM at day eight stained with phalloidin (F-Actin; green) and DAPI (blue). 10x magnification. Scale bar indicates 100 µm. Immunofluorescent confocal images of EVT spheroids without or with ECM at day eight stained with (**d**) HIF1A (green) or (**e**) MMP9 (green), and DAPI (blue). 20x magnification, imaged at center of spheroid. Scale bar indicates 100 µm. Histogram of mean fluorescent intensity of FITC normalized to area of DAPI-positive nuclei for (**f**) HIF1A and (**g**) MMP9. Significant differences between groups determined by unpaired t-test; n = 3. Significant differences between means were indicated by *(p < 0.05).

Considering that the spheroids expressed increased markers of invasion and angiogenesis, we next investigated their actual, functional invasiveness. We adapted and applied a novel, high-throughput spheroid invasion assay for use with our EVT spheroids^17^. Spheroids were embedded into ECM on day two, and observable invadopodia-like projections began to sprout from the spheroid core on day four, increasing in invaded area over the eight days (Fig. 6a). Mean spheroid invasion significantly increased over time, reaching approximately 60–70% invasion at day eight (p < 0.05; Fig. 6b). Using F-actin and DAPI immunofluorescent staining, the invadopodia-like projections were found to be actual cells moving away from the spheroid core and into the ECM (Fig. 6c). Control spheroids that were not embedded in ECM did not exhibit any invadopodia-like projections and remained as round spheres over the eight days (Fig. 6c). ECM-embedded, invasive spheroids further demonstrated significantly increased immunofluorescent staining for HIF1A protein (p < 0.05; Fig. 6d,f) and MMP9 protein (p < 0.05; Fig. 6e,g) compared to control spheroids.

### Spheroid invasion impacted by exogenous drugs and compounds

**Figure 7:**
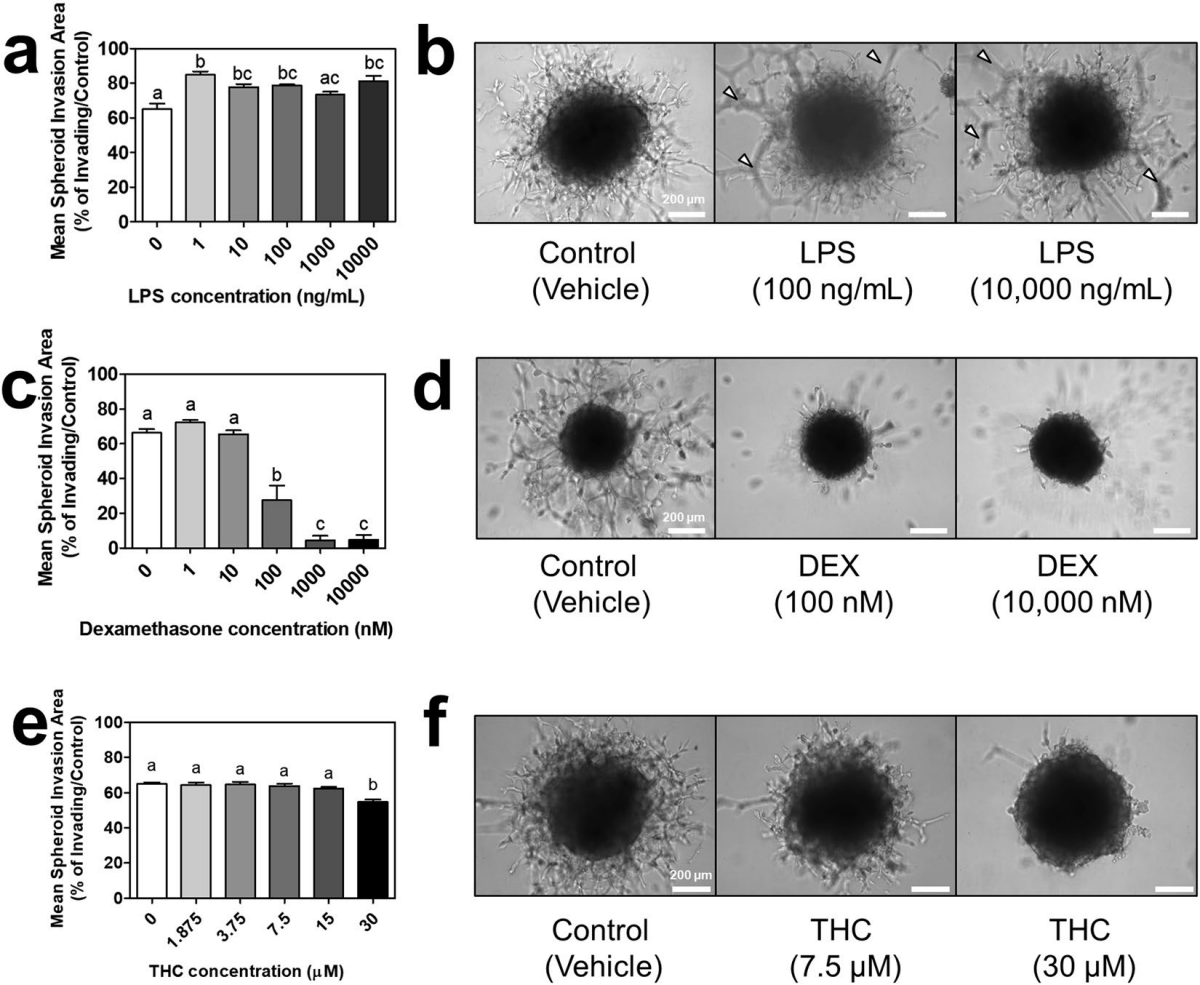
Impact of lipopolysaccharide, dexamethasone, or Δ^9^ – tetrahydrocannabinol on spheroid invasion. (**a**) Mean spheroid invasion areas of EVT spheroids treated with 1–10,000 ng/mL lipopolysaccharide (LPS) on day eight. (**b**) Brightfield images of EVT spheroids in ECM treated with the vehicle control, 100 ng/mL LPS, or 10,000 ng/mL LPS (three representative doses selected along curve to demonstrate effect). White arrows indicate examples of tube formation of EVTs that have invaded to surface of ECM. (**c**) Mean spheroid invasion areas of EVT spheroids treated with 1–10,000 nM dexamethasone (DEX) on day eight. (**d**) Brightfield images of EVT spheroids in ECM treated with the vehicle control, 100 nM DEX, or 10,000 nM DEX. (**e**) Mean spheroid invasion areas of EVT spheroids treated with 1.875–30 µM Δ^9^ – tetrahydrocannabinol (THC) on day eight. (**f**) Brightfield images of EVT spheroids in ECM treated with the vehicle control, 7.5 µM THC, or 30 µM THC. Significant differences between groups determined by one-way ANOVA followed by Tukey’s post-test; n ≥ 3. Significant differences between means as determined by post-tests were indicated by different letters. Images taken at 10x objective magnification. Scale bar represents 200 µm.

Lastly, we were interested in testing the responsiveness of our 3D EVT spheroids to exogenous drugs and compounds. Spheroids were embedded into ECM and treated with lipopolysaccharide, dexamethasone, or THC every 48 hours for up to eight days. Lipopolysaccharide and dexamethasone have previously been shown to augment and inhibit placental invasion, respectively, thus would serve to validate the spheroids’ responsiveness^18–22^. Lipopolysaccharide (1–100 ng/mL and 10,000 ng/mL) indeed significantly augmented EVT invasion by day eight compared to the vehicle control (p < 0.05; Fig. 7a,b). Tube formation was observable for some LPS-treated spheroids, indicating the invasion of EVTs had extended up to the top of the ECM layer (Fig. 7b). Dexamethasone (100–10,000 nM) significantly inhibited EVT invasion by day eight compared to the vehicle control (p < 0.05; Fig. 7c,d). Given the dynamic invasive response to the validated compounds, we were interested in testing the effects of THC, a controversial, less-understood compound with early evidence to impact placental invasion^23^. THC (30 µM) significantly inhibited EVT invasion by day eight compared to the vehicle control (p < 0.05; Fig. 7e,f).

## Discussion

Three-dimensional spheroid and organoid cultures carry the potential to restore *in vivo-*like phenotypes in many *in vitro* organ systems^7^. Our study is the first to generate a specific 3D spheroid *in vitro* model of the trophoblast column that is capable of functional and responsive EVT invasion. We further demonstrate that 3D EVT spheroids: (1) express global transcriptomes that are distinct compared to 2D monolayers, (2) exhibit invasive and migratory gene and protein expression profiles alongside dynamic invasive behaviour when embedded into an ECM environment, and (3) may be studied by adapting the innovative spheroid invasion assay, which was originally pioneered by tumour researchers^17,24^, revealing responsiveness to exogenous drugs. Moreover, the simplicity of our ultra-low attachment microplate approach, continuous real-time monitoring capability, relative low cost of materials required, and use of a well-established cell line, collectively provides an accessible and versatile tool for researchers.

The major transcriptomic differences found in the GSEA and GO analyses altogether suggest that spheroid culturing conditions alone are sufficient to drive EVTs towards expressing a unique genetic profile compared to monolayers. Spheroids possessed enhanced expression profiles of EMT, cell-cell contact, tight junction formation, invasion, and angiogenesis, suggesting they may be a useful model to study EVT invasion. The initial detachment of EVTs from the trophoblast column and continual, but highly-measured, taxis into the maternal decidua is a key phenomenon observed *in vivo* that is not emulated in traditional invasion assays^25^. One major advantage of our model is the establishment of a solid spheroid core, which mimics the microarchitecture and physiochemical driving forces present at the trophoblast column of the anchoring chorionic villi *in vivo* (*e*.*g*., hypoxia/oxygen gradients, mechanical stimulation via ECM, etc.) Only EVTs that are truly invasive would be capable of detaching, invading, and migrating away from the core, whereas traditional assays use individualized cells that are primed to invade via serum-deprivation/reintroduction^25^. HIF1α, a protein known to be rapidly depleted by oxygen, was indeed augmented in our invading spheroids, suggesting the potential involvement of a hypoxia-driven mechanism^26–28^. HIF1α is a key regulator of invasion *in vivo*, and mice with *Hif1α* knocked out experience impaired placental invasion and angiogenesis compared to controls^29^. MMP9, a crucial matrix metalloproteinase in EVT invasion and known to be stimulated by hypoxia^30^, was also augmented at the protein level in invading spheroids. This reveals a preliminary mechanism that is consistent with *in vivo* EVT invasion, although more work must be done to further validate this.

Spheroid cultures also enable the co-existence of proliferative, quiescent and apoptotic states within the same population due to the nature of the 3D construct and the varying depths that cells reside from nutrient and oxygen sources, which better resembles the heterogeneous cellular organization present *in vivo*^7,24^. Accordingly, it has been well-demonstrated by cancer researchers that tumour spheroids exhibit a more physiological response and resistance in drug studies compared to monolayers^24,31,32^, and thus serve as more predictive *in vitro* models. Yang, *et al*.^33^ showed that pancreatic tumour cells cultured as spheroids exhibited greater invasive and migratory behaviour and more consistently formed tumours when injected into nude mice, compared to parental cells cultured as monolayers. Moreover, following paclitaxel chemotherapy treatment, ovarian cancer spheroids remained 20% more viable and proliferative than monolayers, which resembles a chemoresistance similarly seen in other tumours *in vivo*^32^. Following this hypothesis, our 3D EVT spheroids should also provide more a predictive response and resistance to drug exposure. For obvious ethical reasons, it was challenging to directly compare the drug responses of clinical populations given the lack of available data in pregnant women. We do, however, present novel findings that EVT spheroids are responsive to several exogenous compounds, with augmented invasion caused by lipopolysaccharide and inhibited invasion caused by dexamethasone and THC. It is well-documented that lipopolysaccharide increases cytokines like IL-6^34,35^, which in turn stimulate MMP-2/9 activity to induce invasion^18,19^; whereas dexamethasone induces the opposite effect to inhibit invasion *in vivo* and *in vitro*^20,21^. While our results with lipopolysaccharide and dexamethasone are consistent with Librach, *et al*.^22^, there are some contrasting reports in literature that demonstrate an opposite or null effect^34–36^. This may be explained in part by the length of exposure, in which our model enables chronic exposures (eight days) compared to transwell invasion assays that must be acute (24–48 hours)^34–36^. However, this may also reveal a pitfall of transwell invasion assays as a whole, given that these studies do report a consistent downstream effect of the drug (*e*.*g*., increase and decrease in IL-6 via lipopolysaccharide and dexamethasone, respectively), but are simply unable to elicit the expected EVT invasive response^34,35^. Due to the volatility of the inflammatory response and longer-term drug exposures typically observed in clinical situations, it may be argued that our model’s chronic exposures with real-time measurements are necessary to accurately study the impact of drugs on invasion *in vitro*. Lastly, significant inhibition of EVT invasion by the highest dose of THC seen in our work is consistent with *in vitro* findings by Chang, *et al*.^23^, and may reveal a potential mechanism underlying the detrimental impact of this controversial, recreational and medicinal drug on placental and fetal development^23,37^.

While drug screening applications remain limited, our knowledge of stemness and differentiation in placental spheroids and organoids was recently advanced by some exciting studies^9,11,12^. Nandi, *et al*.^9^ demonstrated that HTR8/SVneo and primary placental cells cultured as spheroids may self-renew and differentiate down various trophoblast lineages (*e*.*g*., syncytiotrophoblast). Using Matrigel^®^ drops and organoid media, Turco, *et al*.^11^ cultured primary cytotrophoblasts as organoids that shared high levels of transcriptomic and methylomic similarities with first trimester placental villi, and may also differentiate into invasive, HLA-G-expressing EVTs. Similarly, Haider, *et al*.^12^ used Matrigel^®^ and organoid media to culture primary cytotrophoblasts as stem-like organoids with comparable gene expression profiles as primary cytotrophoblasts and the capacity to differentiate. Together with our current findings, there is accumulating evidence to support 3D culture as a useful and multifaceted model system for placental research.

In conclusion, we provide comprehensive rationale and evidence supporting the development of a microplate-based, spheroid invasion model for placental research. With enhanced invasive and angiogenic capability compared to 2D monolayer cultures and a dynamic response to exogenous compounds, we propose that our model has the potential to be further developed into a high-throughput, screening tool to preclinically assess the safety and impact of drugs and toxins on placental invasion. Given our current lack of knowledge surrounding the effects of both prescribed and illicit drug use in pregnant women, especially in Canada and the United States^38^, there is a great necessity to advance the development of alternative placental models.

## Methods

### Cell culture

Human placental EVT-derived HTR8/SVneo cells (generously provided by Dr. Peeyush Lala, Western University) were used due to the highly-conserved characteristics and gene expression profiles with placental EVTs^39,40^. HTR8/SVneo cells were cultured at 37 °C in a humidified atmosphere of 5% CO_2_/95% atmospheric air in RPMI-1640 (Corning), supplemented with 5% heat-inactivated FBS (Gibco), 1% (v/v) penicillin-streptomycin (Corning) and 1% (v/v) L-glutamine (Corning).

### EVT spheroid generation

96-well ultra-low attachment (ULA) plates (Corning) were used to promote the self-assembly of HTR8/SVneo cells into three-dimensional (3D) cellular spheroids. HTR8/SVneo cells were seeded at 1,000 cells/mL, 5,000 cells/mL and 10,000 cells/mL into the ultra-low attachment plates. Media was replaced every 48 hours post-seeding by aspirating 100 µL of existing media, being careful to not disturb the spheroid, and dispensing 100 µL of fresh media into each well.

### Live cell imaging and analysis of spheroid size

Live images of the spheroids were captured every 48 hours post-seeding at either 4X or 10X objective magnification using an Eclipse Ti-E Inverted Microscope (Nikon). Diameter (µm) of each spheroid was quantified using a manual measurement tool on ImageJ Fiji software (National Institute of Health). Average spheroid diameters were used to generate a linear regression and slope to track the growth over time (µm/day).

### Cell viability staining assay

To confirm cell viability in spheroids over the experimental period, spheroids were incubated with calcein AM (Thermo Fisher; 1:100) for green fluorescence (live cells) and ethidium homodimer-1 (Thermo Fisher; 1:100) for red fluorescence (dead cells) for 30 minutes on days 4, 6 and 8. Spheroids were imaged in 5 µm z-stacks to capture all layers throughout the spheroid using an Eclipse Ti-E Inverted Microscope (Nikon), and all steps were compiled as a z-projection image. Images were analyzed using NIS Elements software (Nikon) to quantify the total areas covered by live or dead cells. The ratio of live to dead cells was then determined by dividing either live or dead cell area over the total area of the spheroid.

### Hematoxylin and eosin (H&E) staining

Spheroids were fixed in 10% formalin within the ULA plates for 15 min at room temperature. An equal volume of liquified 2% agar was added to each well, mixed, and solidified on ice for 20 min. Solid agar pellets with spheroids were transferred into tissue cassettes, placed into 10% formalin for 48 hours, and stored in 70% ethanol. Agar pellets were then embedded in paraffin wax, sliced into 4 µm sections, stained with H&E, and imaged using an Eclipse Ti-E Inverted Microscope (Nikon).

### Transcriptome-wide microarray

Total RNA was extracted from cells using TRIzol Reagent (Invitrogen) and Direct-zol RNA MiniPrep Kit (Zymo Research), following the manufacturer’s protocol. RNA quality was assessed using the RNA 6000 Nano LabChip Bioanalyzer and TapeStation (Agilent), and 200 ng of RNA was processed to assess 20,000+ genes using the Clarion S Human microarray (Thermo Fisher) and GeneChip™ 3000 instrument system (Thermo Fisher), all carried out at the SickKids TCAG Microarray facility.

### Transcriptomic analysis

Normalization and output file generation was performed with Affymetrix Gene Expression Console (Thermo) using the Robust Multi-Chip Analysis (RMA) algorithm [RMA-Gene-full]^41^. Dendrogram was obtained by using all genes available on the platform excluding the control probes with the built-in *hclust* function in R. The height distance to convergence suggests the degree of similarity between the individual branches (samples). The similarity between the branches was measured based on the expression patterns exhibited by the genes in these samples.

Differential expression analysis was performed by using *limma* package in R^42,43^. Genes with FDR p-value < 0.05 were considered to be significantly regulated. Volcano plot was created using the *limma* package as well. Genes regulated with the absolute fold change ≥2 were used for further analyses. Examination and visualization of the significantly over-represented biological processes (Gene Ontology component) was performed by using the BINGO plugin in Cytoscape environment^44^. Gene Set Enrichment Analysis was performed by using C2, C5 and HALLMARK MSigDB collections on whole gene expression profiles^45,46^. Obtained results were further analyzed and visualized by using the EnrichmentMap plugin in Cytoscape environment^47^.

### Real-time quantitative polymerase chain reaction (RT-qPCR)

Total RNA (500 ng) was further reserve-transcribed to cDNA as previously described^8^. Primer sets directed against gene targets of interest were designed through National Center for Biotechnology Information’s Primer-BLAST primer designing tool and synthesized at McMaster’s Mobix Labs (Table 1). Quantitative analysis of mRNA expression was performed via qPCR using SsoAdvanced™ Universal SYBR® Green Supermix (BioRad) and CFX384 Touch Real-Time PCR Detection System (BioRad). The cycling conditions were 95 °C for 10 min, followed by 40 cycles of 95 °C for 10 secs and 60 °C for 10 secs and 72 °C for 15 secs. Relative fold changes were calculated using the comparative cycle times (Ct) method, normalizing all values to the geometric mean of three endogenous control genes (*18S*, *ACTB*, *GAPDH*). The endogenous control gene was selected based on experimentally-determined Ct stability across all treatment groups. Given that all primer sets had equal priming efficiency, the ΔCt values for each primer set were calibrated to the average of all control Ct values, and the relative abundance of each primer set compared with calibrator was determined by the formula 2^ΔΔCt^, in which ΔΔCt was the normalized value.

**Table 1:** Forward and reverse sequences for the primers used for RT-qPCR.

Gene	Forward	Reverse	GenBank
*18S* (*RNA18S5*)	CACGCCAGTACAAGATCCCA	AAGTGACGCAGCCCTCTATG	NR_003286.2
*ACTB*	TTACAGGAAGTCCCTTGCCATC	GCAATGCTATCACCTCCCCTG	NM_001101.5
*GAPDH*	TCACCATCTTCCAGGAGCGA	ATGACGAACATGGGGGCATC	NM_001357943.1
*HIF1A*	CAGCAACGACACAGAAACTGA	TTGGGTGAGGGGAGCATTAC	AF208487.1
*VEGFA*	GGCAGAATCATCACGAAGTGG	GGTCTCGATTGGATGGCAGT	NM_001171623.1
*VEGFC*	CCAATCACACTTCCTGCCGA	GCCTGACACTGTGGTAGTGTT	NM_005429.4
*WNT5A*	CTTGAGCACGACGAAGCAAC	GGAGGTTGGAGACAAAGGGG	NM_003392.4
*TGFB1*	CTCCCGCAAAGACTTTTCCC	GAATAGGGGATCTGTGGCAGG	NM_000660.7
*TGFB2*	TGCACCATGCTTTGGCTTTC	CTGGCTGGCTCAGCAACTAT	NM_001135599.3
*CDH1*	ACACTGGTGCCATTTCCACT	TTAGGGCTGTGTACGTGCTG	AH006175.2
*OCLN*	TCGACCAATGCTCTCTCAGC	CTCCTGGAGGAGAGGTCCAT	U49184.1
*TJP1*	TCTGAGCCTGTAAGAGAGGACT	GCTTGCTGCTTACCTGTTGAG	AF169196.1
*ITGA2*	CGGTTATTCAGGCTCACCGA	ACCTACCAAGAGCACGTCTG	NM_002203.4
*ITGA5*	CCAAAAGAAGCCCCCAGCTA	TCCTTGTGTGGCATCTGTCC	NM_002205.4
*ITGB1*	AAGCGAAGGCATCCCTGAAA	GTCTACCAACACGCCCTTCA	NM_002211.4
*VIM*	GCAAAGACAGGCTTTAGCGAG	TTCAAGTCTCAGCGGGCTC	NM_003380.5
*CLDN1*	CTGTCATTGGGGGTGCGATA	CTGGCATTGACTGGGGTCAT	NM_021101.5
*CLDN4*	CCACTCGGACAACTTCCCAA	ACTTCCGTCCCTCCCCAATA	NM_001305.4
*MMP9*	CCGGCATTCAGGGAGACGCC	TGGAACCACGACGCCCTTGC	NM_004994.2
*TIMP2*	GAAGAGCCTGAACCACAGGT	GGGGGAGGAGATGTAGCAC	NM_003255.4

### Protein extraction and western blot

Total protein was extracted from cells using RIPA buffer supplemented with protease and phosphatase inhibitor cocktails (Roche). The solution was sonicated for 5 sec total, 1 sec per pulse, vortexed, and quantified by colorimetric DC protein assay (BioRad). Loading samples were prepared from fresh total protein extract, Laemelli Sample Buffer (4X) (BioRad) and β-mercaptoethanol, and heated at 90 °C for 5 min to denature the proteins. Proteins (10 μg/well) were separated by size via gel electrophoresis in Mini-PROTEAN® TGX Stain-Free™ 4–20% polyacrylamide gels (BioRad), and transferred onto polyvinylidene difluoride membranes using the Trans-Blot® Turbo™ Transfer System (BioRad). Membranes were blocked in 1x Tris-buffered saline-Tween 20 buffer with 5% non-fat milk, and then probed using anti-MMP9 (Abcam; ab76003; rabbit; 1:500) and anti-TIMP2 (GeneTex; GTX16392; rabbit; 1:500) antibodies diluted in the blocking solution. Donkey anti-rabbit (1:5,000) secondary antibody was used to detect the species-specific portion of the primary antibody, diluted in the blocking solution. Immuno-reactive bands were visualized using Clarity™ Western ECL Substrate (BioRad). Total protein was stained on the membrane using Amido Black and imaged to ensure even loading and transfer^48^.

### EVT spheroid invasion assay

EVT spheroid invasion was studied by embedding the spheroids in an extracellular matrix (ECM) inside the ultra-low attachment plate. Growth-factor reduced Geltrex^®^ (Thermo) was used as the ECM cocktail. On day 2, after the spheroids have self-assembled inside the ULA plate, 100 µL of media was aspirated from each well and replaced with 100 µL of Geltrex^®^ (10 mg/mL) on ice^30^. The entire plate was then centrifuged at 300 g for 3 min at 4 °C to center the spheroids at a consistent position inside the well, and incubated for 60 minutes at 37 °C to allow the ECM to solidify. 100 µL of media was added on top of the solidified ECM. Brightfield images of the control and ECM-embedded spheroids were captured on days 2, 4, 6 and 8 at 4x or 10x objective magnification using an Eclipse Ti-E Inverted Microscope (Nikon) and analyzed using NIS Elements software (Nikon). Area of invasive edge of each spheroid was quantified at each time point by subtracting the average area of corresponding control spheroids from area of the invaded spheroid. Spheroid invasion percentage was then calculated as the ratio of invasive edge area to total invaded spheroid area and used as a relative measure to compare the invasiveness between spheroids^17^.

### Immunofluorescence

Spheroids were fixed overnight in 4% paraformaldehyde and permeabilized for 5 minutes with 0.1% Triton X-100 in PBS. Samples were then blocked for 1 hour using 0.01% Tween-20, 10% goat serum and 1% bovine serum albumin (BSA) in PBS. Afterwards, samples were incubated with HIF1 alpha primary antibody (Abcam; ab179483; rabbit monoclonal; 1:50) or MMP9 primary antibody (Abcam; ab76003; rabbit monoclonal; 1:250) overnight at 4 degrees, and then incubated with Goat Anti-Rabbit IgG H&L Alexa Fluor® 488 secondary antibody (Abcam; ab150077; goat polyclonal; 2 µg/mL) for 2 hours, as necessary. For F-actin staining, samples were incubated with CytoPainter Phalloidin-iFluor 555 reagent (Abcam; ab176756; 1:1000) for 2 hours. Samples were counterstained with 4′,6-diamidino-2-phenylindole dihydrochloride (DAPI; Santa Cruz; 1.5 µg/mL). All blocking and incubations were performed at room temperature, unless otherwise stated. Spheroids were visualized using an Eclipse Ti-E Inverted Confocal Microscope (Nikon). Z-stack images were taken in 10 µm steps to capture all layers and compiled as a z-projection image. Images were analyzed using NIS Elements software (Nikon).

### Drug treatments

Lipopolysaccharide (*Escherichia coli* serotype 055:B5; Sigma Aldrich) stock solutions were prepared from a dried powder at a concentration of 1 mg/mL in phosphate-buffered saline (Corning), and diluted to working concentrations of 1–10,000 ng/mL in RPMI-1640 media (Corning) at time of use. Dexamethasone (Sigma Aldrich) stock solutions were prepared from a dried powder at a concentration of 1 mg/mL in 90% ethanol, and diluted to working concentrations of 1–10,000 nM in RPMI-1640 media at time of use. Δ^9^ – Tetrahydrocannabinol (THC) stock solutions (Sigma Aldrich) were diluted to working concentrations of 1.875–30 µM in RPMI-1640 at time of use. Cells were treated with 100 µL of media containing either the vehicle control or drug treatment every 48 hours for up to eight days.

### Statistical analyses

Prism 6 software (GraphPad) was used to statistically analyze all experimental results. Results were expressed as means of the normalized values ± standard error of the mean (SEM). Data sets were assessed for statistical significance (p < 0.05) through either an unpaired t-test, or one-way analysis of variance (ANOVA), and if significant, Tukey’s *post hoc* test to perform multiple comparisons.

## Supplementary information

Supplementary Information

Supplementary Table S3

Supplementary Table S6

Supplementary Table S9

## Data Availability

All data generated or analyzed during this study are included in this published article (and its Supplementary Information files). Original dataset files are available in the Gene Expression Omnibus (GEO) repository [GEO accession number: GSE126844; https://www.ncbi.nlm.nih.gov/geo/query/acc.cgi?acc = GSE126844].

## References

[1] Burton GJ, Fowden AL (2015). The placenta: a multifaceted, transient organ. Philosophical transactions of the Royal Society of London. Series B, Biological sciences.

[2] Huppertz B, Ghosh D, Sengupta J (2014). An integrative view on the physiology of human early placental villi. Prog Biophys Mol Biol.

[3] Red-Horse K (2004). Trophoblast differentiation during embryo implantation and formation of the maternal-fetal interface. The Journal of clinical investigation.

[4] Knofler M (2010). Critical growth factors and signalling pathways controlling human trophoblast invasion. Int J Dev Biol.

[5] Knofler M, Pollheimer J (2013). Human placental trophoblast invasion and differentiation: a particular focus on Wnt signaling. Front Genet.

[6] Chakraborty C, Gleeson LM, McKinnon T, Lala PK (2002). Regulation of human trophoblast migration and invasiveness. Can J Physiol Pharmacol.

[7] Fennema E, Rivron N, Rouwkema J, van Blitterswijk C, de Boer J (2013). Spheroid culture as a tool for creating 3D complex tissues. Trends Biotechnol.

[8] Wong MK (2018). Extracellular matrix surface regulates self-assembly of three-dimensional placental trophoblast spheroids. PloS one.

[9] Nandi P, Lim H, Torres-Garcia EJ, Lala PK (2018). Human trophoblast stem cell self-renewal and differentiation: Role of decorin. Scientific reports.

[10] Rai A, Cross JC (2015). Three-dimensional cultures of trophoblast stem cells autonomously develop vascular-like spaces lined by trophoblast giant cells. Dev Biol.

[11] Turco MY (2018). Trophoblast organoids as a model for maternal-fetal interactions during human placentation. Nature.

[12] Haider S (2018). Self-Renewing Trophoblast Organoids Recapitulate the Developmental Program of the Early Human Placenta. Stem Cell Reports.

[13] Pijnenborg R, Dixon G, Robertson WB, Brosens I (1980). Trophoblastic invasion of human decidua from 8 to 18 weeks of pregnancy. Placenta.

[14] Hirschhaeuser F (2010). Multicellular tumor spheroids: an underestimated tool is catching up again. J Biotechnol.

[15] Lee Y (2007). A unifying concept of trophoblastic differentiation and malignancy defined by biomarker expression. Hum Pathol.

[16] Davies JE (2016). Epithelial-mesenchymal transition during extravillous trophoblast differentiation. Cell Adh Migr.

[17] Vinci, M., Box, C. & Eccles, S. A. Three-dimensional (3D) tumor spheroid invasion assay. *J Vis Exp*, e52686, 10.3791/52686 (2015). 10.3791/52686 PMC4542056 25993495

[18] Meisser A, Cameo P, Islami D, Campana A, Bischof P (1999). Effects of interleukin-6 (IL-6) on cytotrophoblastic cells. Mol Hum Reprod.

[19] Cohen M, Meisser A, Bischof P (2006). Metalloproteinases and human placental invasiveness. Placenta.

[20] Zhou L, Zhang A, Wang K, Zhou Q, Duan T (2015). Folate ameliorates dexamethasone-induced fetal and placental growth restriction potentially via improvement of trophoblast migration. Int J Clin Exp Pathol.

[21] Zhang D (2016). Glucocorticoid exposure in early placentation induces preeclampsia in rats via interfering trophoblast development. Gen Comp Endocrinol.

[22] Librach CL (1994). Interleukin-1 beta regulates human cytotrophoblast metalloproteinase activity and invasion *in vitro*. The Journal of biological chemistry.

[23] Chang X (2017). Suppression of STAT3 Signaling by Delta9-Tetrahydrocannabinol (THC) Induces Trophoblast Dysfunction. Cellular physiology and biochemistry: international journal of experimental cellular physiology, biochemistry, and pharmacology.

[24] Vinci M (2012). Advances in establishment and analysis of three-dimensional tumor spheroid-based functional assays for target validation and drug evaluation. BMC Biol.

[25] James J, Tun W, Clark A (2016). Quantifying trophoblast migration: *In vitro* approaches to address *in vivo* situations. Cell Adh Migr.

[26] Pringle KG, Kind KL, Sferruzzi-Perri AN, Thompson JG, Roberts CT (2010). Beyond oxygen: complex regulation and activity of hypoxia inducible factors in pregnancy. Hum Reprod Update.

[27] Huppertz B, Weiss G, Moser G (2014). Trophoblast invasion and oxygenation of the placenta: measurements versus presumptions. J Reprod Immunol.

[28] Genbacev O, Joslin R, Damsky CH, Polliotti BM, Fisher SJ (1996). Hypoxia alters early gestation human cytotrophoblast differentiation/invasion *in vitro* and models the placental defects that occur in preeclampsia. The Journal of clinical investigation.

[29] Cowden Dahl KD (2005). Hypoxia-inducible factors 1alpha and 2alpha regulate trophoblast differentiation. Molecular and cellular biology.

[30] Wang L, Yu Y, Guan H, Liu T, Qiao C (2015). 67-kDa Laminin receptor contributes to hypoxia-induced migration and invasion of trophoblast-like cells by mediating matrix metalloproteinase-9. Clin Exp Pharmacol Physiol.

[31] Charoen KM, Fallica B, Colson YL, Zaman MH, Grinstaff MW (2014). Embedded multicellular spheroids as a biomimetic 3D cancer model for evaluating drug and drug-device combinations. Biomaterials.

[32] Loessner D (2010). Bioengineered 3D platform to explore cell-ECM interactions and drug resistance of epithelial ovarian cancer cells. Biomaterials.

[33] Yang Z (2018). Transcriptome Profiling of Panc-1 Spheroid Cells with Pancreatic Cancer Stem Cells Properties Cultured by a Novel 3D Semi-Solid System. Cellular physiology and biochemistry: international journal of experimental cellular physiology, biochemistry, and pharmacology.

[34] Li L (2016). Effects of Lipopolysaccharide on Human First Trimester Villous Cytotrophoblast Cell Function *In Vitro*. Biology of reproduction.

[35] Anton L, Brown AG, Parry S, Elovitz MA (2012). Lipopolysaccharide induces cytokine production and decreases extravillous trophoblast invasion through a mitogen-activated protein kinase-mediated pathway: possible mechanisms of first trimester placental dysfunction. Hum Reprod.

[36] Champion H, Innes BA, Robson SC, Lash GE, Bulmer JN (2012). Effects of interleukin-6 on extravillous trophoblast invasion in early human pregnancy. Mol Hum Reprod.

[37] Khare M, Taylor AH, Konje JC, Bell SC (2006). Delta9-tetrahydrocannabinol inhibits cytotrophoblast cell proliferation and modulates gene transcription. Mol Hum Reprod.

[38] Vorstenbosch S, Kant A, Dabekausen Y (2018). The Dutch Pregnancy Drug Register, a Good Idea for Canada?. J Obstet Gynaecol Can.

[39] Weber M, Knoefler I, Schleussner E, Markert UR, Fitzgerald JS (2013). HTR8/SVneo cells display trophoblast progenitor cell-like characteristics indicative of self-renewal, repopulation activity, and expression of “stemness-“ associated transcription factors. Biomed Res Int.

[40] Graham CH (1993). Establishment and characterization of first trimester human trophoblast cells with extended lifespan. Experimental cell research.

[41] Irizarry RA (2003). Exploration, normalization, and summaries of high density oligonucleotide array probe level data. Biostatistics.

[42] Phipson B, Lee S, Majewski IJ, Alexander WS, Smyth GK (2016). Robust Hyperparameter Estimation Protects against Hypervariable Genes and Improves Power to Detect Differential Expression. Ann Appl Stat.

[43] Ritchie ME (2015). limma powers differential expression analyses for RNA-sequencing and microarray studies. Nucleic Acids Res.

[44] Maere S, Heymans K, Kuiper M (2005). BiNGO: a Cytoscape plugin to assess overrepresentation of gene ontology categories in biological networks. Bioinformatics.

[45] Subramanian A (2005). Gene set enrichment analysis: a knowledge-based approach for interpreting genome-wide expression profiles. Proceedings of the National Academy of Sciences of the United States of America.

[46] Mootha VK (2003). PGC-1alpha-responsive genes involved in oxidative phosphorylation are coordinately downregulated in human diabetes. Nat Genet.

[47] Merico D, Isserlin R, Stueker O, Emili A, Bader GD (2010). Enrichment map: a network-based method for gene-set enrichment visualization and interpretation. PloS one.

[48] Aldridge GM, Podrebarac DM, Greenough WT, Weiler IJ (2008). The use of total protein stains as loading controls: An alternative to high-abundance single-protein controls in semi-quantitative immunoblotting. J Neurosci Meth.

