# Supplementary material for: Transcriptomic and functional analyses of 3D placental extravillous trophoblast spheroids
Source: Sci Rep. 2019 Aug 30;9:12607. doi: 10.1038/s41598-019-48816-8 (PMC6717201; doi:10.1038/s41598-019-48816-8)

Transcriptomic and functional analyses of 3D placental extravillous trophoblast spheroids

Supplementary Tables S1-S2, S4-S5, S7-S8
Supplementary Figure S1

Michael K. Wong[1], Mishquatul Wahed[2], Sarah A. Shawky[2], Anna Dvorkin-Gheva[3], Sandeep Raha[1,2]*

[1] Graduate Program in Medical Sciences, McMaster University, Hamilton, Ontario, Canada, L8N 3Z5.
[2] Department of Pediatrics, McMaster University, Hamilton, Ontario, Canada, L8N 3Z5.
[3] McMaster Immunology Research Centre, McMaster University, Hamilton, ON, Canada, L8N 3Z5.

* Corresponding author email: rahas@mcmaster.ca

Supplementary Table S1. Most significantly up-regulated GSEA canonical pathways in EVTs cultured as 3D spheroids.

Module	Category	Canonical Pathway Term	NES	FDR p-value
**23**	Neuroactive ligand-receptor interaction	Olfactory Signaling Pathway	-3.0542696	0
**23**	Neuroactive ligand-receptor interaction	Olfactory Transduction	-2.966659	0
**6**	Neuroactive ligand-receptor interaction	Class A1 Rhodopsin Like Receptors	-2.5756402	0
**16**	Immune processes, diseases, defensins	Defensins	-2.4561152	0
**6**	Neuroactive ligand-receptor interaction	GPCR Ligand Binding	-2.439125	0
**16**	Immune processes, diseases, defensins	Beta Defensins	-2.4266376	0
**6**	Neuroactive ligand-receptor interaction	Neuroactive Ligand Receptor Interaction	-2.3954382	0
**15**	Signaling events	Peptide Ligand Binding Receptors	-2.3515468	0
**7**	Immune processes, diseases, defensins	Chemokine Receptors Bind Chemokines	-2.2149904	0
**6**	Neuroactive ligand-receptor interaction	Amine Ligand Binding Receptors	-2.1915238	9.33E-05
**7**	Immune processes, diseases, defensins	Cytokine-Cytokine Receptor Interaction	-2.1703234	8.48E-05
**7**	Immune processes, diseases, defensins	Cytosolic DNA Sensing Pathway	-2.162791	7.77E-05
**15**	Signaling events	G Alpha I Signaling Events	-2.142522	7.17E-05
**7**	Immune processes, diseases, defensins	Secreted Factors	-2.0940683	2.38E-04
**13**	ECM	ECM Regulators	-2.0885324	2.22E-04

Supplementary Table S2. Most significantly down-regulated GSEA canonical pathways in EVTs cultured as 3D spheroids

Module	Category	Canonical Pathway Term	NES	FDR p-value
**5**	Diseases	Respiratory Electron Transport	2.80283	0
**5**	Diseases	Respiratory Electron Transport ATP Synthesis by Chemiosmotic Coupling and Heat Production by Uncoupling Proteins	2.735257	0
**5**	Diseases	TCA Cycle and Respiratory Electron Transport	2.715474	0
**2**	Transcription, HIV life cycle	RNA Pol II Transcription	2.701939	0
**2**	Transcription, HIV life cycle	Late Phase of HIV Life Cycle	2.638732	0
**0**	Cell cycle, apoptosis	DNA Replication	2.581079	0
**2**	Transcription, HIV life cycle	mRNA Processing	2.567776	0
**2**	Transcription, HIV life cycle	RNA Pol II Pre-Transcription Events	2.557952	0
**0**	Cell cycle, apoptosis	Mitotic M-M/G1 Phases	2.541292	0
**3**	Cell cycle	DNA Repair	2.527595	0
**0**	Cell cycle, apoptosis	Activation of ATR In Response to Replication Stress	2.509505	0
**2**	Transcription, HIV life cycle	Transcription Coupled Nucleotide Excision Repair	2.504607	0
**3**	Cell cycle	Nucleotide Excision Repair	2.501146	0
**3**	Cell cycle	Nucleotide Excision Repair	2.485817	0
**2**	Transcription, HIV life cycle	mRNA Splicing	2.484428	0

Supplementary Table S3. Full list of GSEA canonical pathways in EVTs cultured as 2D monolayers and 3D spheroids.

Please see attached excel document entitled “Supplementary Table S3 - GSEA Canonical Pathway 2D and 3D”

Supplementary Table S4. Most significantly up-regulated GSEA biological processes in EVTs cultured as 3D spheroids

Module	Category	Biological Process Term	NES	FDR p-value
**34**	Perception	Sensory Perception of Chemical Stimulus	-2.9727	0
**9**	Chemotaxis	Chemokine Mediated Signaling Pathway	-2.23976	3.86E-04
**16**	Immune Processes	Positive Regulation of Inflammatory Response	-2.23484	2.58E-04
**n/a**	n/a	Phospholipase C Activating G Protein Coupled Receptor Signaling Pathway	-2.23057	1.93E-04
**39**	Peptide cross linking	Peptide Cross Linking	-2.2077	1.55E-04
**11**	Immune Processes	Positive Regulation of Leukocyte Chemotaxis	-2.20589	1.29E-04
**11**	Immune Processes	Regulation of Leukocyte Chemotaxis	-2.16591	4.43E-04
**11**	Immune Processes	Positive Regulation of Leukocyte Migration	-2.16474	3.88E-04
**5**	Immune Processes	Defense Response to Bacterium	-2.15508	4.31E-04
**17**	Systemic process	Regulation of Neurological System Process	-2.12934	9.29E-04
**11**	Immune Processes	Regulation of Leukocyte Migration	-2.12286	9.85E-04
**36**	Response to zinc	Cellular Response to Zinc Ion	-2.12121	9.03E-04
**16**	Immune Processes	Regulation of Heat Generation	-2.10755	0.001011
**16**	Immune Processes	Positive Regulation of Acute Inflammatory Response	-2.09269	0.001104
**28**	Immune Processes	Natural Killer Cell Activation Involved in Immune Response	-2.09089	0.001185

Supplementary Table S5. Most significantly down-regulated GSEA biological processes in EVTs cultured as 3D spheroids

Module	Category	Biological Process Term	NES	FDR p-value
**25**	Protein complexes	Mitochondrial Translation	3.070361	0
**25**	Protein complexes	Translational Termination	2.97231	0
**25**	Protein complexes	Translational Elongation	2.886832	0
**2**	Metabolism, biosynthesis, catabolism	Electron Transport Chain	2.729035	0
**1**	Cell cycle	Nucleotide Excision Repair	2.713833	0
**2**	Metabolism, biosynthesis, catabolism	Oxidative Phosphorylation	2.685758	0
**1**	Cell cycle	Transcription Coupled Nucleotide Excision Repair	2.676514	0
**2**	Metabolism, biosynthesis, catabolism	Mitochondrial Respiratory Chain Complex I Biogenesis	2.666465	0
**2**	Metabolism, biosynthesis, catabolism	Mitochondrial Respiratory Chain Complex Assembly	2.661326	0
**1**	Cell cycle	DNA-Dependent DNA Replication	2.659598	0
**4**	Transcription	DNA-Templated Transcription Termination	2.611477	0
**2**	Metabolism, biosynthesis, catabolism	Cellular Respiration	2.577143	0
**25**	Protein complexes	Cellular Protein Complex Disassembly	2.53722	0
**0**	Metabolism, biosynthesis, catabolism	tRNA Metabolic Process	2.504275	0
**4**	Transcription	RNA Splicing via Transesterification Reactions	2.479853	1.21E-04

Supplementary Table S6. Full list of GSEA biological processes in EVTs cultured as 2D monolayers and 3D spheroids.

Please see attached excel document entitled “Supplementary Table S6 - GSEA Biological Processes 2D and 3D”

Supplementary Table S7. Most significantly up-regulated GO biological processes in EVTs cultured as 3D spheroids. GO-ID may be used to cross-reference category and term to find full gene list provided in Supplementary Table S7.

Category	GO-ID	Biological Process Term	FDR p-value
Sensory perception	7608	Sensory Perception of Smell	2.97E-65
Sensory perception	7606	Sensory Perception of Chemical Stimulus	1.36E-63
Sensory perception	7600	Sensory Perception	4.10E-48
Transport	50877	Neurological System Process	5.62E-47
Sensory perception	50890	Cognition	3.54E-46
Transport	3008	System Process	9.63E-46
Response to stimulus, would healing	50896	Response to Stimulus	7.50E-40
Transport	32501	Multicellular Organismal Process	1.74E-34
Response to stimulus, would healing	6952	Defense Response	2.11E-13
Response to stimulus, would healing	7626	Locomotory Behavior	1.73E-10
G-protein signaling	7186	G-Protein Coupled Receptor Protein Signaling Pathway	8.83E-10
Response to stimulus, would healing	42330	Taxis	3.71E-09
Response to stimulus, would healing	6935	Chemotaxis	3.71E-09
Transport	40011	Locomotion	8.68E-09
Immune processes, angiogenesis, response to stimulus	32101	Regulation of Response to External Stimulus	4.36E-08

Supplementary Table S8. Most significantly down-regulated GO biological processes in EVTs cultured as 3D spheroids. GO-ID may be used to cross-reference category and term to find full gene list provided in Supplementary Table S7.

Category	GO-ID	Biological Process Term	FDR p-value
Metabolic, biosynthetic, catabolic processes	44237	Cellular Metabolic Process	8.46E-37
Metabolic, biosynthetic, catabolic processes	8152	Metabolic Process	1.16E-24
Transcription, translation, DNA replication	44260	Cellular Macromolecule Metabolic Process	3.94E-23
Metabolic, biosynthetic, catabolic processes	44238	Primary Metabolic Process	1.19E-19
Metabolic, biosynthetic, catabolic processes	34641	Cellular Nitrogen Compound Metabolic Process	1.50E-18
Cell cycle	6996	Organelle Organization	9.17E-18
Metabolic, biosynthetic, catabolic processes	6139	Nucleobase, Nucleoside, Nucleotide and Nucleic Acid Metabolic Process	2.32E-17
Cell cycle	9987	Cellular Process	4.64E-16
Metabolic, biosynthetic, catabolic processes	6807	Nitrogen Compound Metabolic Process	5.82E-16
Transcription, translation, DNA replication	43170	Macromolecule Metabolic Process	1.04E-15
Cell cycle	16043	Cellular Component Organization	9.53E-15
Transcription, translation, DNA replication	90304	Nucleic Acid Metabolic Process	1.05E-14
Metabolic, biosynthetic, catabolic processes	44249	Cellular Biosynthetic Process	1.27E-14
Transcription, translation, DNA replication	44267	Cellular Protein Metabolic Process	4.32E-13
Metabolic, biosynthetic, catabolic processes	9058	Biosynthetic Process	5.54E-13

Supplementary Table S9. Full list of GO biological processes in EVTs cultured as 2D monolayers and 3D spheroids.

Please see attached excel document entitled “Supplementary Table S8 - GO Biological Processes 2D and 3D”

Supplementary Figure S1. Whole blots for Western blots of (a) MMP9 and (b) TIMP2 protein. A single gel was run and protein transferred onto a PVDF membrane. Membrane was then cut below 63kDa and the top half used for the MMP9 blot. Same membrane was cut just above 25 kDa was used for the TIMP2 blot. Remaining, unused portion of membrane was disposed.

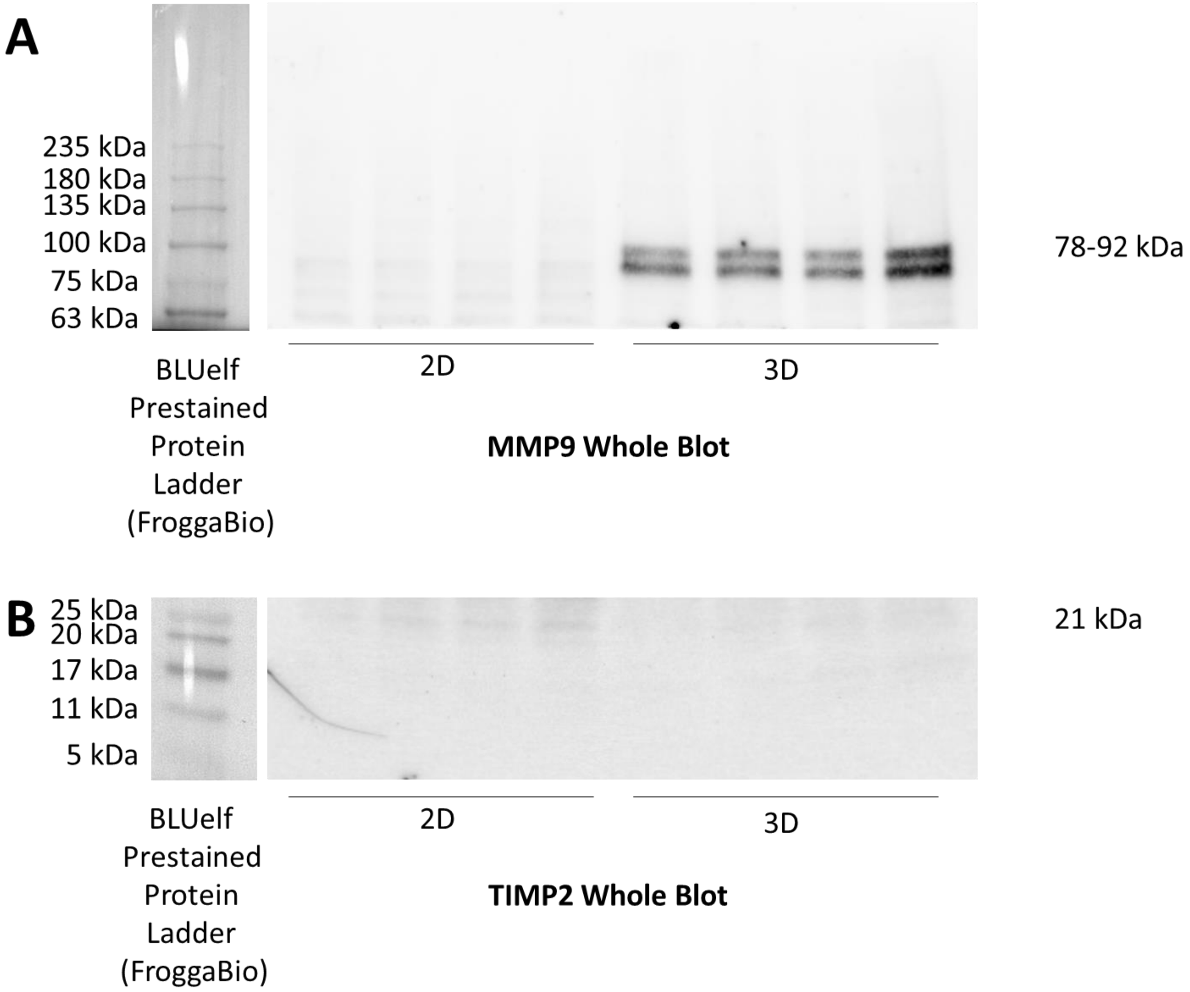

Supplement: Supplementary file 1 — Supplementary Information [file 41598_2019_48816_MOESM1_ESM.pdf]
